# Detection of OXA-181 Carbapenemase in *Shigella flexneri*

**DOI:** 10.3201/eid3005.231558

**Published:** 2024-05

**Authors:** Ghulam Dhabaan, Hassan Jamal, Danielle Ouellette, Sarah Alexander, Karen Arane, Aaron Campigotto, Manal Tadros, Pierre-Philippe Piché-Renaud

**Affiliations:** The Hospital for Sick Children, Toronto, Ontario, Canada (G. Dhabaan, H. Jamal, S. Alexander, K. Arane, A. Campigotto, M. Tadros, P.-P. Piché-Renaud);; University of Toronto, Toronto (G. Dhabaan, H. Jamal, S. Alexander, K. Arane, A. Campigotto, M. Tadros, P.-P. Piché-Renaud);; King Abdulaziz University, Jeddah, Saudi Arabia (H. Jamal);; Western University, London, Ontario (D. Ouellette)

**Keywords:** *Shigella flexneri*, carbapenemase, OXA-48, OXA-48-like, OXA-181, bacteria, antimicrobial resistance, beta-lactamases, enteric infections, Canada, India

## Abstract

We report the detection of OXA-181 carbapenemase in an azithromycin-resistant *Shigella* spp. bacteria in an immunocompromised patient. The emergence of OXA-181 in *Shigella* spp. bacteria raises concerns about the global dissemination of carbapenem resistance in Enterobacterales and its implications for the treatment of infections caused by *Shigella* bacteria.

*Shigella flexnerii* infection leads to shigellosis, an acute gastrointestinal disease. Shigellosis affects socioeconomically disadvantaged and densely populated communities that have unsafe water, poor sanitation, and poor hygiene (*1*). *Shigella* spp. bacteria are major contributors to acute bloody diarrhea worldwide, adding to disease numbers and death in children under 5 years of age (*2*). The emergence of multidrug-resistant *Shigella* strains is a concerning trend. Multidrug-resistant strains resist multiple first-line oral antimicrobials (i.e., ampicillin, trimethoprim/sulfamethoxazole, and ciprofloxacin). The situation is further complicated by enzyme-mediated β-lactam resistance in *Shigella* bacteria, further impacting empiric therapy and making the isolates extensively drug-resistant (*2*). Although extensively drug-resistant isolates have remained susceptible to carbapenem therapy, carbapenem resistance in *Shigella* spp. through imipenemase-type metallo-β-lactamase, New Delhi metallo-β-Lactamase, and Verona integron-encoded metallo-β-lactamase has been reported (*3*,*4*).

We report a case of OXA-181–producing *S. flexneri* bacteria recovered from the stool of an immunocompromised patient with B-cell acute lymphoblastic leukemia (B-ALL). OXA-181 is a subtype of the OXA-48–like carbapenemase enzymes, classified as an Ambler class D β-lactamase, that primarily hydrolyzes penicillins and carbapenems. Those enzymes are usually transmitted on plasmids and are typically associated with Enterobacterales such as *Klebsiella pneumoniae* and *Escherichia coli* bacteria (*5*).

A 2-year-old girl, born in a rural area near Hyderabad, India, was diagnosed with standard-risk B-ALL. Her chemotherapy treatment was complicated by 2 episodes of culture-negative febrile neutropenia and acute gastroenteritis. Her diarrhea was presumed to be allopurinol-induced and was managed with supportive care. Her care team discovered evidence of a B-ALL relapse. The patient recovered from the fever and diarrhea, and her family immigrated to Canada, where the patient was admitted to a hospital to establish care for her relapsed B-ALL. 

The patient was afebrile and did not have diarrhea until day 3 in the hospital, when she had onset of febrile neutropenia, nonbloody diarrhea, and abdominal pain. In accordance with the hospital’s infection prevention protocol, we collected a stool sample for carbapenemase-producing Enterobacterales (CPE) screening. It exhibited growth of non–lactose fermenting colonies on the OXA side of a Chromid Carba Smart plate (bioMérieux, http://www.biomerieux.com), which we confirmed to be *S. flexneri* bacteria type 2a by using a biochemical panel and serotyping. We performed a stool PCR by using Seegene Allplex GI-EB gastrointestinal multiplex assay (SeeGene Inc., https://www.seegene.com) that showed the presence of *Shigella* spp. bacteria and astrovirus. We also isolated *S. flexneri* bacteria from a stool culture by using molecular detection (Appendix).

We began treatment for febrile neutropenia with piperacillin/tazobactam and vancomycin, in addition to azithromycin because of the detection of *S. flexneri* bacteria from the patient stool samples. Both isolates from the CPE screen and stool culture demonstrated a similar susceptibility profile (Table 1). Although the meropenem MIC was susceptible according to Clinical and Laboratory Standards Institute breakpoints, it was higher than 0.12 mg/L, the CPE screening cutoff in our laboratory protocol (*6*). We used the CARBA-5 assay (NG Biotech, https://www.ngbiotech.com) to further evaluate the antibiotic susceptibility, and the results indicated the presence of an OXA-48–like enzyme. The Public Health Ontario Laboratory verified the presence of OXA-48–like gene by using multiplex PCR (*7*). Because of the azithromycin resistance, we modified the treatment to trimethoprim/sulfamethoxazole. After treatment, the patient experienced rapid defervescence and resolution of the diarrhea. We repeated the stool culture after 2 weeks of treatment, and the culture resulted in no growth of *S. flexneri* bacteria.

**Table 1:** Antibiotic-susceptibility results using 5 different methodologies for the *Shigella flexneri* bacteria cultured from an immunocompromised patient, Canada*

Antibiotic	BD Phoenix,† mg/L	Broth microdilution, mg/L	Agar dilution, mg/L	CLSI breakpoints for susceptibility, mg/L	Kirby–Bauer disk diffusion, mm
Azithromycin	NA	NA	>32	<8	NA
Ceftriaxone	0.5	0.5	NA	<1	28
Ceftazidime	0.5	1	NA	<4	30
Ertapenem	>1	1	<0.5	<0.5	24
Meropenem	0.5	0.5‡	<0.12	<1	24
Ciprofloxacin	2	>2	NA	<0.25	NA
TMP/SMX	<0.5	2/38	NA	<2/38	NA
Colistin	NA	NA	<0.25	<2§	NA

*CLSI, Clinical and Laboratory Standards Institute; NA, not available; TMP/SMX, trimethoprim/sulfamethoxazole. †Becton Dickinson, https://www.bd.com. ‡Lowest concentration for meropenem on methodology used (Gram negative sensititer panel). §Intermediate susceptibility.

We conducted whole-genome sequencing (Appendix). We extracted DNA from the bacterial isolate by using easyMag (bioMérieux) and sequenced on a GridION system with a R10.4.1 flow cell (Oxford Nanopore Technologies, https://nanoporetech.com). We analyzed data with MinKNOW 23.04.5 (Oxford Nanopore Technologies) to construct a consensus genome. We analyzed the isolate’s genome and plasmid with the Comprehensive Antibiotic Resistance Database (CARD) (http://arpcard.mcmaster.ca), identifying 5 resistance genes on the plasmid (Table 2), including OXA-181 with >95% identity and length within the plasmid. The plasmid has a size of 91,956 bp and carries all the genes for the resistance profile (Appendix). We deposited the plasmid gene sequence into GenBank (accession no. PP417752).

**Table 2:** Antibiotic-resistance genes detected within the plasmid recovered from a *Shigella flexneri* bacteria cultured from an immunocompromised patient sample, based on the Comprehensive Antibiotic Resistance Database,* Canada

Gene	Phenotypic resistance
OXA-181	Carbapenems
*qnrS1*	Fluoroquinolones
*mrx*	Macrolides
*mphA*	Macrolides
*ermB*	Macrolides

*Available at http://arpcard.mcmaster.ca.

Given the low-hydrolytic activity of OXA-48–like enzymes, microbiology laboratories face difficult challenges in accurately detecting these enzymes in Enterobacterales. The Clinical and Laboratory Standards Institute breakpoints for meropenem are not suited for CPE surveillance, potentially missing OXA-48–like producers (*8*). Our laboratory has adopted a meropenem MIC breakpoint of >0.12 mg/L for CPE screening, in line with European Committee on Antimicrobial Susceptibility Testing recommendations (*9*). This approach is crucial for identifying isolates that require further CPE investigation, especially considering the reduced activity of OXA-48–like enzymes against cephalosporins.

Identification of an OXA-181 carbapenemase in a plasmid carried by *S. flexneri* bacteria is an alarming finding and concerning for the spread of this resistance profile in densely populated low- and middle-income communities. The detection of OXA-181 in *Shigella* spp. bacteria increases concerns about the broad dissemination of carbapenem resistance among other Enterobacterales (*10*). This finding emphasizes the need for vigilant and targeted surveillance for CPE in at-risk patients.

Appendix Additional information on detection of OXA-181 carbapenemase in *Shigella flexneri*.

## References

[1] World Health Organization. Water, Sanitation and Hygiene (WASH) [cited 2024 Feb 26]. https://www.who.int/health-topics/water-sanitation-and-hygiene-wash.32031570

[2] Centers for Disease Control and Prevention Health Alert Network. Increase in extensively drug-resistant shigellosis in the United States [cited 2024 Feb 26]. https://emergency.cdc.gov/han/2023/pdf/CDC_HAN_486.pdf 10.1097/JCMA.0000000000000270 32134861 PMC7153464

[3] Abdelaziz NA. Phenotype-genotype correlations among carbapenem-resistant Enterobacterales recovered from four Egyptian hospitals with the report of SPM carbapenemase. Antimicrob Resist Infect Control. 2022;11:13. 10.1186/s13756-022-01061-7 35063019 PMC8783469

[4] Thamizhmani R, Rhagavan R, Sugunan AP, Vijayachari P. VIM- and IMP-type metallo-β-lactamase-producing *Shigella* spp. in childhood diarrhea from Andaman Islands. Infect Dis (Lond). 2015;47:749–50. 10.3109/23744235.2015.1022874 25768231

[5] Pitout JDD, Peirano G, Kock MM, Strydom KA, Matsumura Y. The global ascendency of OXA-48-type carbapenemases. Clin Microbiol Rev. 2019;33:e00102–19. 10.1128/CMR.00102-19 31722889 PMC6860007

[6] Clinical and Laboratory Standards Institute. Performance standards for antimicrobial susceptibility testing: thirty-third informational supplement (M100-S33). Wayne (PA): The Institute; 2023.

[7] Public Health Ontario. CPE – carbapenemase producing Enterobacteriaceae – Confirmation [cited 2024 Feb 26]. https://www.publichealthontario.ca/en/Laboratory-Services/Test-Information-Index/CPE-confirmation 10.1093/cid/ciaa287 32179890 PMC7184482

[8] Fattouh R, Tijet N, McGeer A, Poutanen SM, Melano RG, Patel SN. What is the appropriate meropenem MIC for screening of carbapenemase-producing Enterobacteriaceae in low-prevalence settings? Antimicrob Agents Chemother. 2015;60:1556–9. 10.1128/AAC.02304-15 26711746 PMC4776007

[9] European Committee on Antimicrobial Susceptibility Testing. EUCAST guideline for the detection of resistance mechanisms and specific resistances of clinical and/or epidemiological importance. Version 2.0. 2017 Jul [cited 2024 Feb 26]. https://www.eucast.org/resistance_mechanisms

[10] Baker S, Scott TA. Antimicrobial-resistant *Shigella*: where do we go next? Nat Rev Microbiol. 2023;21:409–10. 10.1038/s41579-023-00906-1 37188805 PMC10184058

