# Supplementary material for: Detection of OXA-181 Carbapenemase in Shigella flexneri
Source: Emerg Infect Dis. 2024 May;30(5):1048–50. doi: 10.3201/eid3005.231558 (PMC11060442; doi:10.3201/eid3005.231558)
Supplement: Appendix — Additional information on detection of OXA-181 carbapenemase in Shigella flexneri. [file 23-1558-Techapp-s1.pdf]

Article DOI: https://doi.org/10.3201/eid3005.231558

EID cannot ensure accessibility for supplementary materials supplied by authors. Readers who have difficulty accessing supplementary content should contact the authors for assistance.

Detection of OXA-181 Carbapenemase in *Shigella flexneri*

Appendix

Shigella flexneri isolation methodology

From the carbapenemase-producing Enterobacterales (CPE) surveillance specimen, the stool specimen was inoculated on CHROMID® CARBA SMART Agar. The next day, scant gray colonies grew on the OXA side of the plate. The organism was identified as *Escherichia coli* by matrix assisted laser desorption ionization-time of flight (MALDI-TOF) mass spectrometry (Bruker Daltonics). However, because the colonies were gray, not pink on the agar, and due to a known limitation that MALDI-TOF cannot distinguish *E. coli* from *Shigella*, serotyping using *Shigella* antisera and biochemical reactions were set up. Kligler iron agar was alkaline over acid with no H2S and produced little gas. The organism was negative for lysine, ornithine, indole, and citrate. Motility was negative, and the isolate showed agglutination with *S. flexneri* group B antisera, types 1–6, and no agglutination with antisera for other *Shigella* groups. The identification was confirmed by the BD Phoenix system, which gave a 99% *S. flexneri* identification of the isolate. The isolate was forwarded to the Provincial laboratory and was serotyped as *S. flexneri* type 2a. Stool culture ordered as a reflex test after detection of *Shigella* species by multiplex PCR identified the organism when it grew as non-lactose fermenting colonies on MacConkey agar. All biochemical and serotyping results were identical to the isolate from the CPE surveillance specimen described previously.

Isolate whole genome sequencing methodology

DNA was extracted from an overnight culture of the bacterial isolate using the easyMag system (bioMérieux). Purified DNA was prepared for sequencing using the Rapid Barcoding

Sequencing Kit SQK-LSK114 (Oxford Nanopore Technologies, UK). Sequencing occurred on a GridION system using a R10.4.1 flow cell (FLO-MIN114, Oxford Nanopore Technologies) with High-accuracy model basecalling with data captured over 26 hours. Data was evaluated with the MinKNOW software 23.04.5. A consensus genome was constructed using Flye 2.9.

Identification of antibiotic resistance genes

The consensus genome for both the bacterial isolate and plasmid were analyzed through the Resistance Gene Identification tool within the Comprehensive Antibiotic Resistance Database (*1*). Hits demonstrating a ≥95% identity and ≥95% length of reference sequence were included in analysis. This database identified 5 resistance genes present within the plasmid (Appendix Table). The plasmid consensus sequence was visualized and annotated using Geneious Prime v2023.0.1 (Biomaters, U.S.).

Appendix Table. Resistance Gene Identification obtained through the CARD database for the isolated plasmid.

RGI Criteria	ARO Term	% Identify of Matching Region	% Length of Reference Sequence
Perfect	OXA-181	100	100
Perfect	QnrS1	100	100
Perfect	Mrx	100	100
Perfect	MphA	100	100
Strict	ErmB	97.96	98.79

Reference

1. Alcock BP, Huynh W, Chalil R, Smith KW, Raphenya AR, Wlodarski MA, et al. CARD 2023: expanded curation, support for machine learning, and resistome prediction at the Comprehensive Antibiotic Resistance Database. Nucleic Acids Res. 2023;51(D1):D690–9. PubMed https://doi.org/10.1093/nar/gkac920
